# Quantitative analysis of castration resistant prostate cancer progression through phosphoproteome signaling

**DOI:** 10.1186/1471-2407-14-325

**Published:** 2014-05-08

**Authors:** Reynald M Lescarbeau, David L Kaplan

**Affiliations:** 1 Department of Biomedical Engineering, Tufts University, 4 Colby St, Medford, MA 02155, USA

**Keywords:** Prostate cancer, Phosphoproteome, Castration resistance, Regression analysis, Cell signaling

## Abstract

**Background:**

Although recent progress has been made in treating castration resistant prostate cancer, the interplay of signaling pathways which enable castration resistant growth is incompletely understood. A data driven, multivariate approach, was used in this study to predict prostate cancer cell survival based on the phosphorylation levels of key proteins in PC3, LNCaP, and MDA-PCa-2b cell lines in response to EGF, IGF1, IL6, TNFα, dihydrotestosterone, and docetaxel treatment.

**Methods:**

The prostate cancer cell lines were treated with ligands or inhibitors, cell lyates were collected, and the amount of phosphoprotein quantified using 384 well ELISA assays. In separate experiments, relative cell viability was determined using an MTT assay. Normalized data was imported into Matlab where regression analysis was performed.

**Results:**

Based on a linear model developed using partial least squares regression, p-Erk1/2 was found to correlate with castration resistant survival along with p-RPS6, and this model was determined to have a leave-one-out cross validated R^2^ value of 0.61. The effect of androgen on the phosphoproteome was examined, and increases in PI3K related phosphoproteins (p-Akt, p-RPS6, and p-GSK3) were observed which accounted for the majority of the significant increase in androgen-mediated cell survival. Simultaneous inhibition of the PI3K pathway and treatment with androgen resulted in a non-significant increase in survival. Given the strong effect of PI3K related signaling in enabling castration resistant survival, the specific effect of mTor versus complete inhibition was examined using targeted inhibitors. It was determine that mTor inhibition accounts for 52% of the effect of complete PI3K inhibition on cell survival. The differences in signaling between the cell lines were explored it was observed that MDA-PCa-2b exhibited far less activation of p-Erk in response to varying treatments, explaining one of the reasons for the lack of castration resistance.

**Conclusion:**

In this work, regression analysis to the phosphoproteome was used to illustrate the sources of castration resistance between the cell lines including reduced p-Erk signaling in MDA-PCa-2b and variations in p-JNK across the cell lines, as well as studying the signaling pathways which androgen acts through, and determining the response to treatment with targeted inhibitors.

## Background

Every year 223,000 men will be diagnosed with prostate cancer in the United States with most patients having androgen dependent disease at the initial stages [1]. Although there have been recent advances in treating castration resistant prostate cancer, prognoses are still poor once the disease progresses to the castration resistant, metastatic state [2,3]. There have been numerous mechanisms reported which can enable castration resistant growth including intracrine synthesis of androgen, upregulation of the androgen receptor (AR), co-activation of the AR by other pathways, or complete bypass of androgen signaling through the activation of other pathways [4-6]. These mechanisms can include activation of oncogenes, mutation of tumor suppressors, epigenetic alterations, or activation of a pathway through extracellular matrix or ligand cues contained in the microenvironment.

The signaling mechanisms which enable castration resistant growth have been studied using various cell line models, including PC3, LNCaP, and MDA-PCa-2b cells lines. These cell lines display a range of phenotypes, including aggressive castration resistant growth in PC3 cells and androgen-dependent growth in LNCaP and MDA-PCa-2b cells. These cell lines additionally display various mutations in their genome with LNCaP and PC3 cells having inactivated PTEN (Phosphatase and tensin homolog) and MDA-PCa-2b cells having intact PTEN [7,8].These differences are used to model the variation present in patients with differing stages of disease progression. Depending on the cell line, certain growth factor treatments such as EGF (Epidermal growth factor) or IGF1 (Insulin-like growth factor 1), or targeted kinase inhibitor treatments, can enhance castration resistant growth or treat castration resistant cancer through modulating signal transduction pathways.

The analysis of prostate cancer signaling often involves the examination of numerous pathways through genomics, transcriptomics, or proteomics. The relationship of these data sets to cell phenotype is often multivariate and non-intuitive. To investigate these relationships, multivariate linear regression techniques have been utilized over the last decade, and have been successful in correlating the signaling of multiple pathways using phosphoproteomic data to phenotypic outcomes including apoptosis, proliferation, invasion, and migration [9-11]. Partial least squares (PLS) regression is a multiple linear regression algorithm which correlates variation in the Y matrix (cell survival) to the X matrix (phosphoprotein levels) by identifying vectors which simultaneously describe variation in both data sets. These latent variables are able to account for the multicollinearity of similarly regulated phosphoproteins (i.e. phosphosites which may be part of the same pathway or crosstalk between pathways).

In the present work, the objective was to correlate castration resistant growth to pathway activation via phosphoproteomic signaling using regression analysis. The use of the PC3, LNCaP, and MDA-PCa-2b cell lines allowed us to capture diversity in different prostate cancer genotypes, and make comparisons across cell lines. The epigenetic and genetic variations are assumed to be abstracted into the levels of phosphoprotein activation (i.e., PTEN inactivating mutations causing higher levels of p-Akt) with differences in unmeasured pathways across cell lines being sources of error in the model. This approach enables the exploration of a range of hypotheses to understand how cell signaling drives castration resistance, the importance of various signaling proteins in enabling castration resistant growth, the correlation between these signaling proteins, and the specific effect of various targeted kinase inhibitors in modulating the effect of these signaling proteins. This work will aid in the long term goal of optimizing the inhibition of signaling pathways to prevent castration resistant prostate cancer progression.

## Methods

### Cell culture and reagents

LNCaP, MDA-PCa-2b, and PC3 cell lines were acquired from ATCC (Manassas, VA, USA). PC3 and LNCaP cells lines were cultured in 10% fetal bovine serum (FBS), RPMI 1640, and 1% antibiotic-antimycotic. The MDA-PCa-2b cell line was cultured in BRFF-HPC1 media purchased from AthenaES (Baltimore, MD, USA) supplemented with 20% FBS. Dihydrotestosterone was acquired from Sigma-Aldrich (St. Louis, MO, USA). Androgen depleted media consisted of 10% charcoal stripped FBS with phenol red free RPMI 1640. Docetaxel was acquired from Sigma-Aldrich. Temsirolimus and SB202190 were purchased from Selleckchem (Houston, TX, USA). All other inhibitors were purchased from EMD Millipore (Billerica, MA, USA). Unless otherwise stated all other cell culture reagents were acquired from Invitrogen (Grand Island, NY, USA).

### Cell survival assay

Relative cell viability was assessed using an MTT ((3-(4,5-Dimethylthiazol-2-yl)-2,5-diphenyltetrazolium bromide) assay acquired from Invitrogen. As previously determined by our lab, MTT correlates to relative cell number as confirmed via DNA quantification and manual cell counting [12]. All three cell lines were plated at a concentration of 5,000 cells/cm^2^ in a 24 well plate in their respective growth media. The cells were allowed to adhere for 24 hours. The media was then changed to androgen depleted media which the cells were cultured in for an additional 72 hours. Finally, relative cell viability was determined using an MTT assay according to the manufacturer’s instructions. Targeted kinase inhibitors were used at the following concentrations: LY294002 at 7 μm, U0126 at 325 nm, Wedelolactone at 10 μm, Temsirolimus at 50 nm, and SB202190 at 500 nm. Additionally, the total protein amount of biological replicates from each cell type was measured using a Bicinchoninic assay purchased from Thermo Scientific (Rockford, IL, USA). After measuring cell survival with an MTT assay the results were normalized to total protein measured to account for variations in cell size between the cell lines.

### Measuring phosphoprotein levels

Each prostate cancer cell line was plated to six well plates at a density of 7,500 cells/cm^2^ in their respective growth media and allowed to adhere for 24 hours. After 24 hours cells were treated with androgen depleted media supplemented with the appropriate treatment. For studies involving the use of inhibitors on LNCaP cells, the cells were first pretreated for 30 minutes with the inhibitor before additional treatments were added to ensure complete inhibition. Following the appropriate amount of time (30 minutes, 4 hours, or 24 hours) the media was removed and the cells were lysed. R&D Systems (Minneapolis, MN, USA) Duoset ELISA kits were used to quantify the amount of phosphoprotein present in each sample. Lysates were processed and the assays performed according to manufacturer’s instructions. A Bicinchoninic acid assay was performed on each lysate and the lysates were diluted such that 20 ug of protein lysate was used in each ELISA assay. The lysis buffer was made by combining 20 ml of PBS, 1nM of EDTA, 5 mM NaF, 6 M Urea, 0.1 ml of Triton ×100, and 2 packets on Halt protease/phosphatase inhibitors from Thermo Scientific (Waltham, MA, USA). Briefly, the antibody pairs for the ELISA assays were optimized on 384 well ELISA plates from Santa Cruz Biotechnology (Dallas, Texas, USA) using the accompanied positive control samples. An eight point standard curve was generated and fitted using a second order polynomial. The amount of phosphoprotein in ng per 20 ug of total protein lysate was then determined by comparing the measured absorbance of the sample to the standard curve.

### Data analysis

Following data acquisition, calibration to the ELISA standard curve, and normalization to total protein content, the data was imported into Matlab (The Mathworks, Natick, MA) where both protein (X matrix) and survival data (Y matrix) were mean centered and unit variance scaled. The data was arranged such that each column of the X matrix represented a phosphoprotein at a specific time (8 phosphoproteins × 3 time points = 24 columns). The rows represent the cell treatments with the values in the X matrix corresponding to phosphorylation levels and the rows of the Y matrix corresponding to relative cell survival in response to that treatment. The X and Y matrices were then inputted into a function which utilizes the native plsregress function packaged with Matlab to employ the SIMPLS algorithm and calculate the regression coefficients. This was repeated with each row (treatment condition) left out. The calculated model was applied to the left out data to determine a predicted Y value. The R2 value was then calculated using the measured (Y vector) and predicted survival data. Partial least squares regression is a multiple regression algorithm which attempts to explain the Y matrix by finding a multidimensional direction in the X space which explains the maximum variation in both matrices [10]. This algorithm is especially suited to applications where the X matrix contains many more variables than observations, or when many of the X variables are multicollinear, as is often the case in cell signaling data.

An approach for calculating significance in PLS regression models was employed which randomizes the X matrix (phosphoprotein data) as compared to the Y matrix (survival data) and performs regression analysis. From this randomized regression a R^2^ is calculated and saved. We repeated this procedure 3,000 times and determined a mean R^2^ and standard deviation for these calculated random models. The randomized R^2^ values were assumed to follow a normal distribution. Using the mean and standard deviation from the R^2^ values calculated for randomized regression, and the R^2^ of the correctly calculated model, the number of standard deviations away from the random mean was determined (z-score), and from this a p-value determined.

The level of phosphoprotein activation in response to ligand treatment was calculated as a percent increase over untreated controls. This data was imported into Cytoscape and used as relative measures of edge thickness between ligand and the resulting phosphoproteins [13]. Decreases in phosphoprotein levels in response to treatment were depicted as a red edge.

### Correlation modeling

To model the correlation between the phosphosites in the three different cell lines, the Pearson correlation between all possible unique pairs of phosphosites within the same cell line were assessed and a P-value calculated which represents the statistical significance of the correlation. This was completed on observations for untreated cells, EGF, IGF1, IL6, TNFα, DHT, and docetaxel treated cells using all three time points (30 minutes, 4 hours, and 24 hours). The Q-value (P-value equivalent adjusted for multiple hypothesis testing) was also determined using the Q-value software downloaded from the Storey lab website to adjust for multiple hypothesis testing [14].

## Results

### Measuring phosphorylation and castration resistant survival in response to treatment

**Figure 1:**
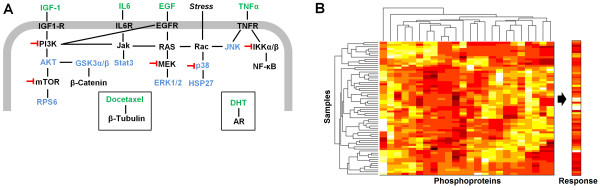
**An overview of the measured signaling pathways and responses to treatment. A)** A diagrammatic overview illustrating the cell signaling between the phosphoproteins measured (blue lettering), the treatments used (green lettering), and the inhibitors which were used on the LNCaP cells (red lines). **B)** Heatmap with hierarchical clustering illustrating the mean centered and variance scaled (z-score) changes in phosphoprotein values in response to varying treatments (see Methods), as well as survival values of LNCaP, PC3, or MDA-PCa-2b cells as measured via a MTT assay.

To obtain a diverse response across multiple phosphosites in LNCaP, PC3, and MDA-PCa-2b cells, the cells were treated with the ligands EGF (Epidermal growth factor), IGF1 (insulin-like growth factor 1), IL6, TNFα (Tumor necrosis factor alpha), dihydrotestosterone (DHT) which is an androgen receptor agonist, and the chemotherapeutic docetaxel. LNCaP cells were additionally treated with the targeted kinase inhibitors LY294002 (Phosphoinositide 3-kinase (PI3K) inhibitor), U0126 (Mitogen-activated protein kinase kinase (MEK) inhibitor), wedelactone (IκB Kinase (IKK) α/β inhibitor), temsirolimus (Mammalian target of rapamycin (mTOR) inhibitor), and SB202190 (p38 inhibitor), each in combination with the previously mentioned ligands (Additional file 1: Table S4). These ligands and drugs were selected because of their involvement in moderating prostate signaling pathways which have been implicated in castration resistant growth of prostate cancer, as well as their availability and characterized activity. Whole cell lysates were collected at 30 minutes, 4 hours, and 24 hours post treatment and assayed using 384 well plate phospho-ELISA assays to measure the response of phosphorylation sites in key pathways to treatment with these ligands and inhibitors. In the signaling pathways diagram, a simplistic representation of the interactions between the measured phosphoproteins, the pathways which contain those proteins, and the effect of the targeted inhibitors can be observed (Figure 1A). The phosphosites which were measured in response to treatment are listed (see Phosphosites measured). These particular phosphosites were selected based on an examination of the literature, and their potential to enable cell growth in androgen depleted conditions.

Phosphosites measured

Erk1: T202/Y204 [15]

Erk2: T185/Y187

Akt1: S473 [15,16]

Akt2: S474

Akt3: S472

RPS6: S235/S236 [16]

GSK3α: S21 [17]

GSk3β: S9

p38δ: T180/Y182 [18]

JNK1 and JNK2: T183/Y185 [19]

JNK3: T221/Y223

HSP27: S78/S82 [20]

Stat3: Y705 [21]

After the phosphoprotein data was collected and normalized (see methods), hierarchical clustering analysis was applied across the phosphosites at the three time points as well as the treatment groups. This analysis measures the similarity between each observation using a Euclidean distance metric (Figure 1B). Across the y dimension of the X matrix, the treatments were found to cluster first by cell line and then by inhibitor treatment (for LNCaP cells only), with little clustering in the ligand treatment groups (Figure 1B and Additional file 2: Table S1). In the x dimension the phosphoprotein activation was generally found to cluster the three time points of each phosphoprotein together (Figure 1B and Additional file 3: Table S2). This clustering indicated that the cell line, and then inhibitor, and finally the ligand treatment imparted the most substantial changes in the cells in the y dimension (treatment conditions). In the × dimension (phosphoproteins), the data indicated that the change by time point tended to cause the most substantial response in phosphoprotein levels.

For each treatment, biological duplicates were measured and the absolute percentage difference between the two replicates was determined (Additional file 4: Table S3). A mean difference of 20.4% was observed across all cell lines which when compared to the finding that the phosphosites varied by approximately 670% on average over untreated controls, was considered an acceptable amount of error.

### Regression analysis correlating phosphoprotein measurements to cell survival in androgen depleted conditions

**Figure 2:**
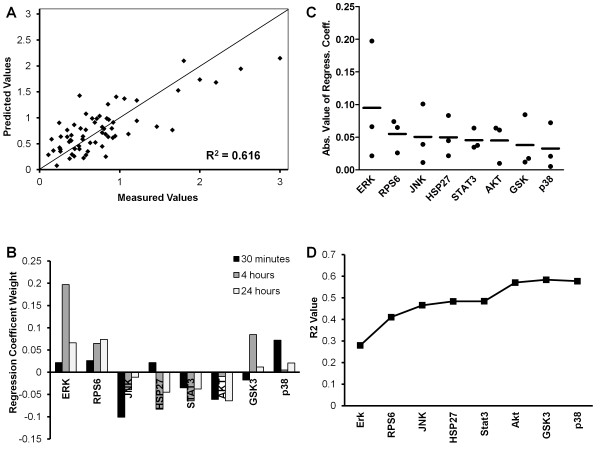
**Partial least squares regression results with three principal components. A)** A scatter plot illustrating the measured versus predicted values for each different treatment group across all three cell lines (LNCaP, PC3, MDA-PCa-2b). **B)** The regression coefficients for all 8 phosphoproteins across the 3 time points. **C)** The mean absolute value of the regression coefficients, indicating the contribution of that phosphoprotein to the overall model. **D)** The R-squared value as additional phosphoproteins are added to a 3 principal component partial least squares regression model. The R-squared value of Erk is for a model built on Erk data alone. The R-squared value for RPS6 is for a model built on Erk and RPS6 data. The R-squared value for p38 is for the complete model for all data and is 0.577.

In an attempt to understand how the alterations in signaling may lead to variations in survival outcomes in cells grown in androgen depleted conditions, we built a statistical model using PLS regression. The data was arranged so that the phosphoprotein data was regressed against the survival data using PLS regression on the complete data set of 8 phosphoproteins, at 3 time points, using 3 cell lines, with 6 treatments. After calculating the model parameters the leave-one-out cross validated R^2^ value was determined to be 0.616 with 3 latent variables, and the predicted versus measured survival values were plotted (Figure 2A). Additional latent variables beyond three had marginal explanatory power due to the fact that the majority of the variation in the X matrix could be described in terms of these latent variables, therefore three components were used for all analyses. When this calculated R^2^ value was compared to the mean R^2^ value calculated from randomized models (X matrix rows randomized against Y matrix rows) we observed that this model was 6.36 standard deviations above the mean randomized value of 0.1847 corresponding to a P-value less than 0.0001. This result indicates that this model can correlate to survival significantly better than by random chance.

Upon determining that this model was significantly more accurate than a randomized model, we examined the regression coefficients to determine weights calculated on the different phosphoproteins. Consistently positive coefficients for p-Erk (Extracellular signal-regulated kinases) were noted, as well as consistently increased p-RPS6 (Ribosomal protein S6) across all time points (Figure 2B). p-JNK regression coefficients were negative at all time points along with p-Akt and p-Stat3. p-GSK3 (Glycogen synthase kinase 3) additionally had minimal early and late time point regression coefficients, however had a substantially increased 4 hour regression coefficient.

In order to better assess the contribution of the regression coefficients to the model outcome the absolute value of the coefficients was taken for each time point and the mean plotted for each phosphoprotein in descending order (Figure 2C). From this, p-Erk was determined to most strongly contribute to the model, followed by p-RPS6 and p-JNK. We used this data to plot the R^2^ value of models built on increasing amounts of data, starting with p-Erk and adding phosphoproteins in order of their mean absolute value of regression coefficients. It can be seen that a model built solely on p-Erk, p-RPS6, and p-JNK resulted in R^2^ values of 0.4655 as compared to the complete model which gave us a R^2^ value of 0.616 (Figure 2D). Beyond these phosphoproteins, only the Akt phosphoprotein added substantial further information to the model, increasing the R^2^ from 0.484 to 0.570, indicating this data added substantial accuracy to the model without having a large regression coefficient. From these results it was concluded that the phosphorylation levels of Erk, RPS6, JNK, and Akt were able to explain the majority of variation in castration resistant survival across these three cell lines.

The amount of error between the predicted values from the model and the measured values were also grouped by treatment, cell line, and inhibitor (for LNCaP cells treated in combination with targeted inhibitors) (Additional file 5: Figure S1A, B, and C). The only significant difference that was observed between any conditions was a much higher docetaxel error (Additional file 5: Figure S1A). This is likely due to the fact that docetaxel is a chemotherapeutic which causes cell death, however little variation in the phosphoproteome as compared to controls was seen. Therefore a model of phosphoproteomic signaling was unable to predict docetaxel’s apoptotic effect.

### The effect of androgen treatment on phosphoprotein signaling

**Figure 3:**
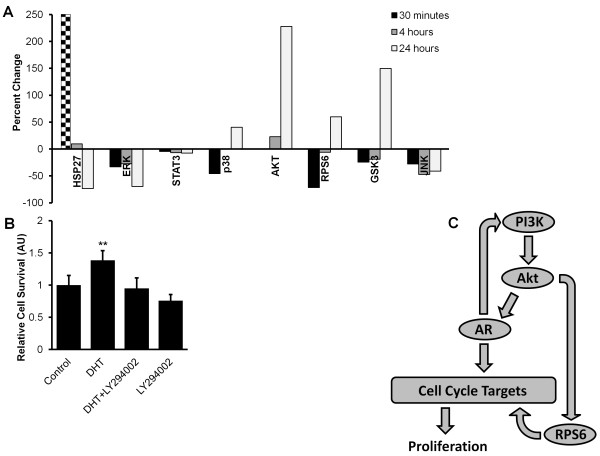
**Modeling the effect of androgen treatment of cell signaling. A)** The percent change in phosphoprotein levels due to DHT treatment of LNCaP cells in androgen depleted media as compared to the control condition. A HSP27 phosphorylation value of 3200% was observed at the 30 minute time point. **B)** The relative survival of LNCaP cells under various treatment conditions in response to androgen treatment (DHT), a PI3K inhibitor (LY294002), or a combination of DHT and LY294002 in androgen depleted media as compared to the control condition of androgen depleted media. DHT is significantly greater than all other groups (** equals P-value < 0.01). Error bars are std. dev. from mean. Values are normalized to the untreated control condition’s mean. **C)**A diagrammatic overview of the proposed signaling interactions between the androgen receptor, PI3K signaling pathway, RPS6, and cell cycle targets.

The effect of DHT on phosphoprotein activation was examined across the different treatments conditions. Previous research indicates that the activated AR may act through growth factor pathways such as PI3K (Phosphoinositide 3-kinase), and by causing the transcription of genes which may directly activate the cell cycle [22]. Upon examining the DHT treatment group an increase in the 24 hour p-RPS6 and p-Akt levels as compared to controls was observed in LNCaP cells (Figure 3A). The effect of DHT on PC3 and MDA-PCa-2b cells was also examined. PC3 cells exhibited no substantial alterations in signaling which is consistent with previous reports where PC3 cells had minimal to no AR expression [23]. MDA-PCa-2b cells exhibited an increase in p-RPS6 and p-GSK3β at 4 hours which was not maintained through 24 hours, although DHT treatment of MDA-PCa-2b cells did not cause survival increases to the extent that EGF or IGF1 treatment did.

The survival of LNCaP cells in response to DHT treatment was examined and an increase of 38% was observed as compared to the control condition (Figure 3B). This survival advantage was completely abrogated when treated in combination with LY294002 (PI3Ki) which reduced p-Akt, p-GSk3, and p-RPS6 to below baseline levels at all time points. The combination of DHT plus LY294002 caused a non-significant increase in survival of 25% over the treatment of LY294002. There was little difference in phosphoprotein levels from LY294002 treatment alone, indicating direct activation of the cell cycle by AR or activation of other non-measured pathways by AR other than PI3K.

Based on these observations we propose a modification of the model originally proposed by Gosh et al. (Figure 3C) [24]. Here, the PI3K pathway can activate the AR which can activate the cell cycle. However, activation of the AR can also activate the PI3K pathway. Additionally, activation of the PI3K pathway can activate cell cycle through bypassing the AR via mTOR/RPS6.

### Comparison of phosphoprotein alterations between LNCaP, MDA-PCa-2b, and PC3 cell lines

**Figure 4:**
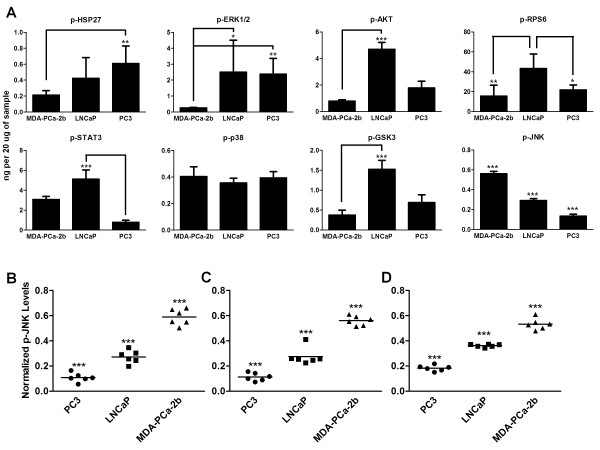
**The mean phosphorylation value for each phosphoprotein across the three cell lines. A)** These phosphoprotein levels were averaged from the EGF, IGF, IL6, TNFα, DHT, and docetaxel treatment in androgen depleted media for each cell line. **B)** JNK phosphorylation values at 30 minutes for all three cell lines. **C)** JNK phosphorylation values at 4 hours for all three cell lines. **D)** JNK phosphorylation values at 24 hours for all three cell lines. In **B**, **C**, and **D** all groups are significantly different from each other (* equals P-value < 0.05, ** equals P-value < 0.01, and *** equals P-value < 0.001).

The differences between the signaling of the three different cell lines used were examined by taking the mean phosphoprotein level across all treatments, with the exception of inhibitor treatments in LNCaP cells. Several observations were noted in this data including the consistent trend across p-Akt, p-RPS6, and p-GSK3 of higher values in the LNCaP cells, somewhat reduced values in the PC3 cells, and the lowest amount of phosphoprotein in MDA-PCa-2b cells (Figure 4A). These phosphosites are part of the PI3K pathway which likely explains their similar levels of activation (the measured GSK3 phosphosites of GSK3α at S21 and GSK3β at S9 are activated by p-Akt). When p-Erk levels were measured in MDA-PCa-2b cells, consistently lower amounts of this phosphoprotein were found as compared to LNCaP and PC3 cells (10.7% of LNCaP levels and 11.3% of PC3 levels, Figure 4A). Based on the substantial weight placed on the p-Erk regression coefficient, this explains one of the major reasons for reduced castration resistance in MDA-PCa-2b cells.

A final observation made regarding the mean phosphoprotein levels across all treatments was the decreasing levels of phosphorylation in JNK from MDA-PCa-2b cells to LNCaPs and then PC3 cells (Figure 4B, C, and D). Initially, this was a counterintuitive observation due to the fact that this phosphosite has previously been described as an oncogene, and we have measured castration resistance in the cell lines inverse to the amount of p-JNK (Additional file 6: Figure S2) [25]. However, this observation corroborates recent work indicating that JNK acts as an oncogene in tumor development and a tumor suppressor in regards to castration resistant growth [19].

**Figure 5:**
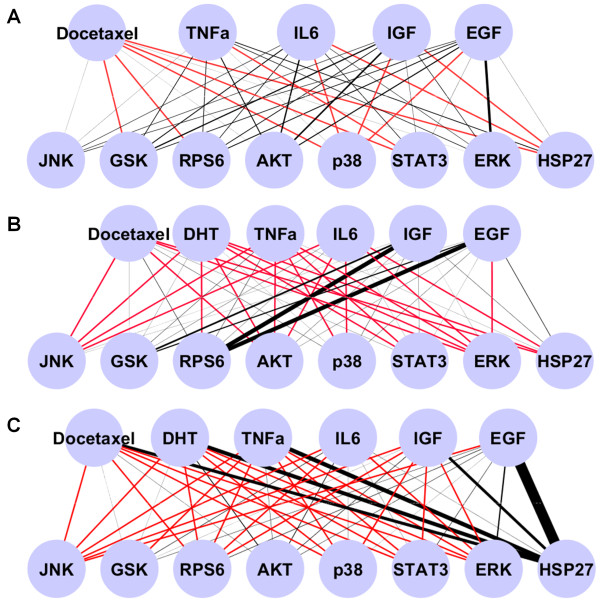
**The relative activation of each phosphoprotein induced by each ligand treatment.** Line thickness is proportional to percent increase over untreated control of cells in androgen depleted media. Red lines indicate a reduction in phosphoprotein levels. **A)** The activation of phosphoproteins in PC3 cells in response to ligand treatment. Red lines indicate a reduction in phosphoprotein levels. **B)** The activation of phosphoproteins in MDA-PCa-2b cells in response to ligand treatment. **C)** The activation of phosphoproteins in LNCaP cells in response to ligand treatment.

In order to better illustrate the activation of phosphoproteins between cell lines in response to treatments, graphs were created which plot the phosphoprotein response as a function of edge thickness (Figure 5A, B, and C). Upon examining these graphs substantial variation between the cell lines is observed with the most castration resistant cell line, PC3, having the weakest response generally to the various treatments, followed by moderate responses in LNCaP cells, and strong sensitivity to certain growth factors in MDA-PCa-2b cells. Furthermore, there were differences between the cell lines in response to the same growth factor. In PC3 and LNCaP cells EGF stimulates Erk to various extents, however in MDA-PCa-2b cells EGF had little effect on Erk and strongly increased p-RPS6 along with IGF1 which was not seen to have an effect LNCaP or PC3 cells.

### Modeling the effect of treatments and targeted inhibitors

**Figure 6:**
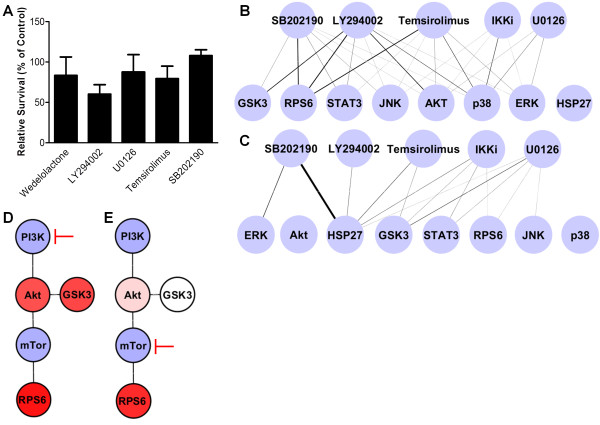
**Response of LNCaP cells to targeted inhibitors. A)** The relative cell survival of LNCaPs treated with targeted inhibitors in androgen depleted media after 72 hours. Relative survival was normalized to untreated control LNCaPs in androgen depleted media (100%). Error bars are std. dev. from mean. **B)** The degree to which each targeted inhibitor reduced phosphorylation of the phosphoprotein as compare to control conditions. A thicker line indicates a stronger reduction in phosphorylation. Edges in average effect over 30 minutes, 4 hours, and 24 hours. **C)** The degree to which each targeted inhibitor activated phosphorylation of the phosphoprotein as compare to control conditions. A thicker line indicates an increase phosphorylation. Edges in average effect over 30 minutes, 4 hours, and 24 hours. **D)** The effect of LY294002 treatment on p-Akt, p-GSK3, and p-RPS6. PI3K and mTor were not measured. A stronger color red indicates increased inhibition. **E)** The effect of Temsirolimus treatment on p-Akt, p-GSK3, and p-RPS6. PI3K and mTor were not measured. A stronger color red indicates increased inhibition.

The effect of treatment with five targeted kinase inhibitors on protein phosphorylation and the LNCaP cell survival in androgen depleted media as compared to controls can be seen (Figure 6A, B, and C). Cells were treated with concentrations five times the published IC50 (half maximal inhibitory concentration) values of the target kinases which, assuming a hill coefficient of one, is equal to IC83. Some of the targeted kinase inhibitors did not reduce their target phosphoproteins to the anticipated levels, possibly due to degradation. Incomplete inhibition of targets should have no effect on model performance because the response is predicted according to actual measured phosphoprotein levels. We calculated a separate PLS regression model solely on all of the LNCaP data, including inhibitor treatments. A leave-one-out cross valuidated R^2^ value of 0.58 (Additional file 7: Figure S3) was observed across this data set indicating that the response from inhibitor treatment can predict the majority of the variation in cell survival.

The effect of complete PI3K inhibition with LY294002 versus mTor inhibition alone with temsirolimus was also examined. Based on the relative survival levels of LNCaP cells treated with LY294002 versus temsirolimus it was determined that the temsirolimus treated group had 31% increased cell survival over cells treated with LY294002. However, both treatments reduced the p-RPS6 to similar levels which were near complete inhibition from basal levels, while LY294002 also strongly reduced measured p-Akt and p-GSK3 levels (Figure 6D and 6E). Based on this observation it was concluded that signaling upstream of mTor (such as p-GSK3 which was observed to be highly correlated to p-Akt) accounted for the difference in survival between complete PI3K inhibition and inhibition of mTor alone.

### Modeling the correlation between phosphosites’ activation

**Figure 7:**
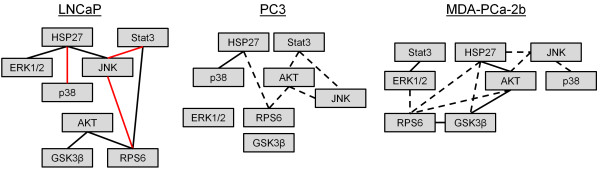
**The correlation values between phosphoproteins across the three different cell lines.** Black lines indicate a positive correlation while red lines indicate a negative correlation. Solid lines indicate statistical significance when adjusted for multiple hypothesis testing while dotted lines indicate a P-value of less than 0.05 before multiple hypothesis correction.

In order to better understand the correlation between different phosphoproteins’ activation under the same treatment we examined the Pearson correlation between them across the three separate cell lines (for LNCaP cells the inhibitor plus treatment data was excluded). The most consistent theme across the cell lines was the positive correlation between p-RPS6 and p-Akt, which occurs through mTor (Q-value of 0.0531, 0.0391, and 0.0160, for PC3, LNCaP, and MDA-PCa-2b cells, respectively, Figure 7). Additionally, there was a correlation between p-Akt and p-GSK3 present in LNCaP cells (Q-value of 0.00569) and MDA-PCa-2b cells (Q-value of 0.000216), but not PC3 cells (Q-value of 0.42972).

## Discussion

The goal of this work was to examine how variation in disparate signaling pathways altered castration resistant growth of three different prostate cancer cell lines in response to activating treatments and targeted inhibitors. In future work, an understanding of how multiple signaling pathways enable castration resistance in patients will be critical to optimizing patient specific treatments using targeted therapies. Differences in the basal level of castration resistant growth across the three cell lines were observed, as was their response to the treatments. A regression model was developed for predicting castration resistant growth and survival, using an MTT assay, which far exceeded randomized data sets (P-value < 0.0001), and was able to account for over half of the variation in cell survival (R^2^ = 0.616). The MTT assay acted as an approximate metric of cell survival and abstracted the proliferation and apoptosis balance as well as other cellular processes such as neuroendocrine differentiation into one value representing total cell survival in androgen depleted conditions in response to treatment. There are numerous other pathways which are perturbed in prostate cancer by the treatments used here, as well as epigenetic and genetic variability which likely account for the remaining unexplained variance in cell survival, however a majority of cell survival can be explained by these 8 phosphoproteins’ activation level at three time points.

When the effect of androgen treatment on phosphoproteomic signaling was examined we observed an increase in PI3K related phosphoprotein activation (p-Akt, p-RPS6, p-GSK3) at later time points. This is consistent with the observation that AR activation can cause activation of the PI3K pathway, at least in part, through induction of IGF1 secretion [6]. Previous work has indicated that activation of the PI3K pathway can coactivate the AR, causing reciprocal feedback [26]. Additionally, the AR can cause the transcription of cell cycle-related genes directly through binding to the promoter elements and transcribing genes such as c-Myc [27].

Phosphoprotein levels across cell lines were also examined and there was a clear inverse trend between innate castration resistance and p-JNK levels which did not substantially vary in response to treatment. As previously discussed, this effect may play a role in castration resistance [19]. This variation between cell lines was also seen in the lack of consistent correlation between phosphosites indicating that the genetic and epigenetic differences between the cell lines significantly alters how cell signaling networks respond to treatment. PI3K-related signaling was the only exception to this which had somewhat conserved correlation values across cell lines.

To make additional comparisons PLS regression was performed on the individual cell line data yielding models of cell survival with high R-squared values (LNCaP: R^2^ = 0.986, PC3: R^2^ = 0.998, and MDA-PCa-2b: R^2^ = 0.969). Upon examining the regression coefficients from these models PC3 cells generally weighted positively p-Erk, p-Stat3, p-RPS6, and p-GSK3 as compared to LNCaP which generally weighted p-Erk, p-Stat3, and p-GSK3 positively. Finally, MDA-PCa-2b weighted positively p-Akt, p-RPS6, and p-GSK3 in determining cell survival (Additional file 8: Figure S4). From this data it can be seen that survival appears to be largely mediated through PI3K-related signaling in MDA-PCa-2b cells with an increasing role of p-Erk and p-Stat3 in LNCaP and PC3 cells. Additionally, given the few preserved correlations observed across all cell lines, the data indicates that variations between cell lines cause substantial changes in signaling crosstalk.

The use of targeted kinase inhibitors allowed the elucidation of the role of particular phosphoproteins. Specifically, we identified the role of phosphoproteins upstream of mTor in the PI3K in enabling survival. In a recent phase II clinical trial Temsirolimus as a single agent had an effect on 32% of patients, and numerous PI3K inhibitors are being investigated for use in prostate cancer [28]. Additionally, an increase in p-Erk was noted in response to treatment with the p38 inhibitor SB202190 which is consistent with the observation that p38 inhibition can increase survival [29,30].

## Conclusion

In this work, regression analysis was used to determine how cell signaling correlates with castration resistant growth across three cell lines. Based on the data presented, *in vitro* prostate cancer cell androgen independent growth could be largely described via MAPK (Mitogen-activated protein kinases) and PI3K signaling. Androgen mediated signaling also largely acted through PI3K signaling. p-JNK appeared to potentially play a role in the fundamental castration resistance of a cell line, and MDA-PCa-2b cells did not utilize p-Erk to enable androgen-independent growth. Given the myriad of targeted inhibitors currently in development, approaches similar to this work which determine drivers of disease progression may enable optimizing treatment given the unique signaling of each patient.

## Abbreviations

AR: Androgen receptor; DHT: Dihydrotestosterone; EGF: Epidermal growth factor; Erk: Extracellular signal-regulated kinases; GSK3: Glycogen synthase kinase 3; IC50: Half maximal inhibitory concentration; IGF1: Insulin-like growth factor 1; IKK: IκB Kinase; JNK: c-Jun N-terminal kinases; MAPK: Mitogen-activated protein kinases; MEK: Mitogen-activated protein kinase kinase; mTor: Mammalian target of rapamycin; PI3K: Phosphoinositide 3-kinase; PLS: Partial least squares; PTEN: Phosphatase and tensin homolog; RPS6: Ribosomal protein S6; TNFα: Tumor necrosis factor alpha.

## Competing interests

The authors declare they have no competing interests.

## Authors’ contributions

RML designed and implemented the experimental procedures. DLK oversaw experimental procedure, and DLK and RML wrote the manuscript. All authors read and approved the final manuscript.

## Pre-publication history

The pre-publication history for this paper can be accessed here:

http://www.biomedcentral.com/1471-2407/14/325/prepub

## Supplementary Material

Additional file 1: Table S4 Phosphoprotein and survival data for all cell lines under ligand and inhibitor treatment. Phosphoprotein values are the average of two biological replicates and are expressed as ng per 20 ug of protein lysate. Survival values are normalized measurements of cell survival based on an MTT assay as described in the methods. Click here for file

Additional file 2: Table S1 Mean percent error between duplicate phosphoprotein measurements. Click here for file

Additional file 3: Table S2 Row labels for the hierarchical clustering heat map. Row names are in the order as they are presented on the heat map in Figure 1B with the first row name representing the top row in the heat map. Click here for file

Additional file 4: Table S3 Column labels for the hierarchical clustering heat map. Column names are in order as presented on the heat map in Figure 1B with the first column name representing the left most column. Click here for file

Additional file 5: Figure S1 Percent error of the model between measured and predicted survival values across different variables. A) The absolute percent difference between the measured and predicted survival values for each treatment. The error for the docetaxel treatment group is significantly different from all other treatment groups (*** equals a P-value < 0.001). B) The absolute percent difference between the measured and predicted survival values grouped by inhibitor treatment on LNCaP cells. C) The absolute percent difference between the measured and predicted survival values as grouped by cell line. Click here for file

Additional file 6: Figure S2 Survival of PC3, LNCaP, and MDA-PCa-2b cells in androgen depleted media. Cell survival in androgen depleted conditions as compared to the normal growth media condition as measured with an MTT assay. One hundred percent is equal to the mean of that cell line in culture with its normal growth media. All pairs of groups were significant from each other at the 95% confidence interval (*** equals P-values < 0.001. Click here for file

Additional file 7: Figure S3 The measured versus predicted survival of LNCaP cells. The predicted versus measured survival of LNCaP cells treated with targeted kinase inhibitors in combination with ligand treatments and docetaxel. A R^2^ value of 0.58 was calculated based on the partial least squares regression performed. Click here for file

Additional file 8: Figure S4 The weights of the regression coefficients from PLSR for each cell line calculated individually. The regression coefficient’s weights for three different partial least squares regression models constructed on the data for the LNCaP, PC3, or MDA-PCa-2b cell lines when treated with EGF, IGF1, IL6, TNFα, DHT, and docetaxel. Click here for file
