# Supplementary figures and images for: Quantitative analysis of castration resistant prostate cancer progression through phosphoproteome signaling
Source: BMC Cancer. 2014 May 8;14:325. doi: 10.1186/1471-2407-14-325 (PMC4031492; doi:10.1186/1471-2407-14-325)

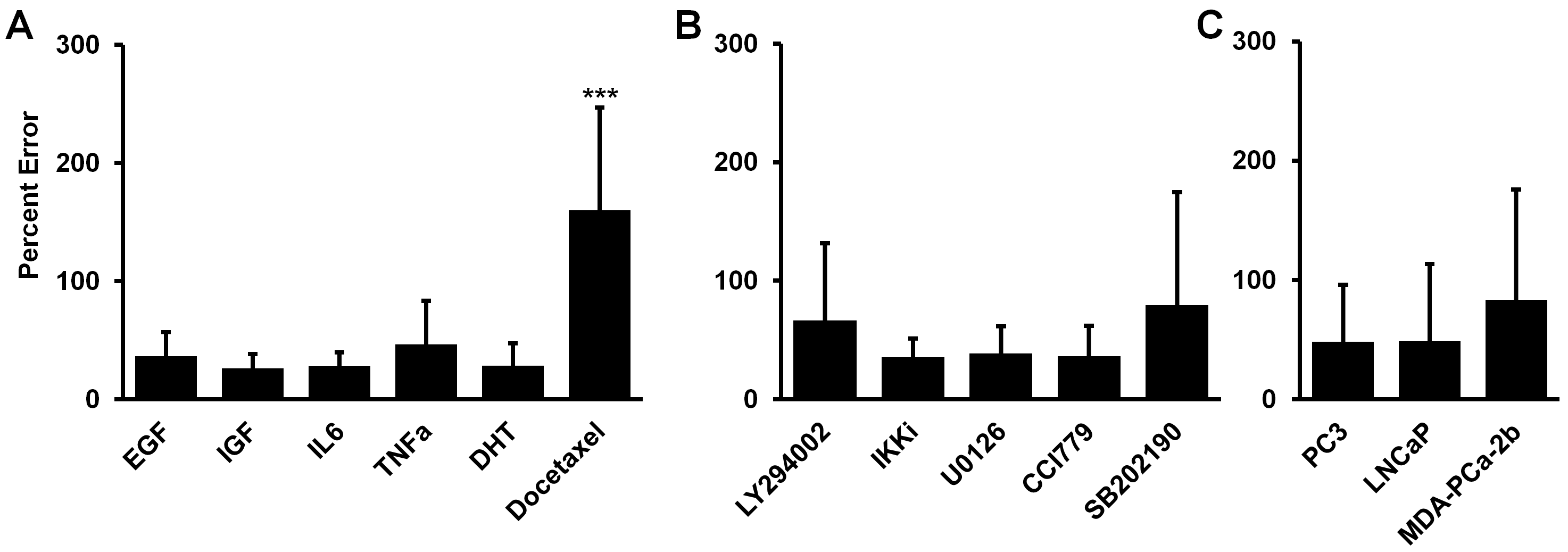

Supplement: Additional file 5: Figure S1 — Percent error of the model between measured and predicted survival values across different variables. A) The absolute percent difference between the measured and predicted survival values for each treatment. The error for the docetaxel treatment group is significantly different from all other treatment groups (*** equals a P-value < 0.001). B) The absolute percent difference between the measured and predicted survival values grouped by inhibitor treatment on LNCaP cells. C) The absolute percent difference between the measured and predicted survival values as grouped by cell line. [file 1471-2407-14-325-S5.tiff]

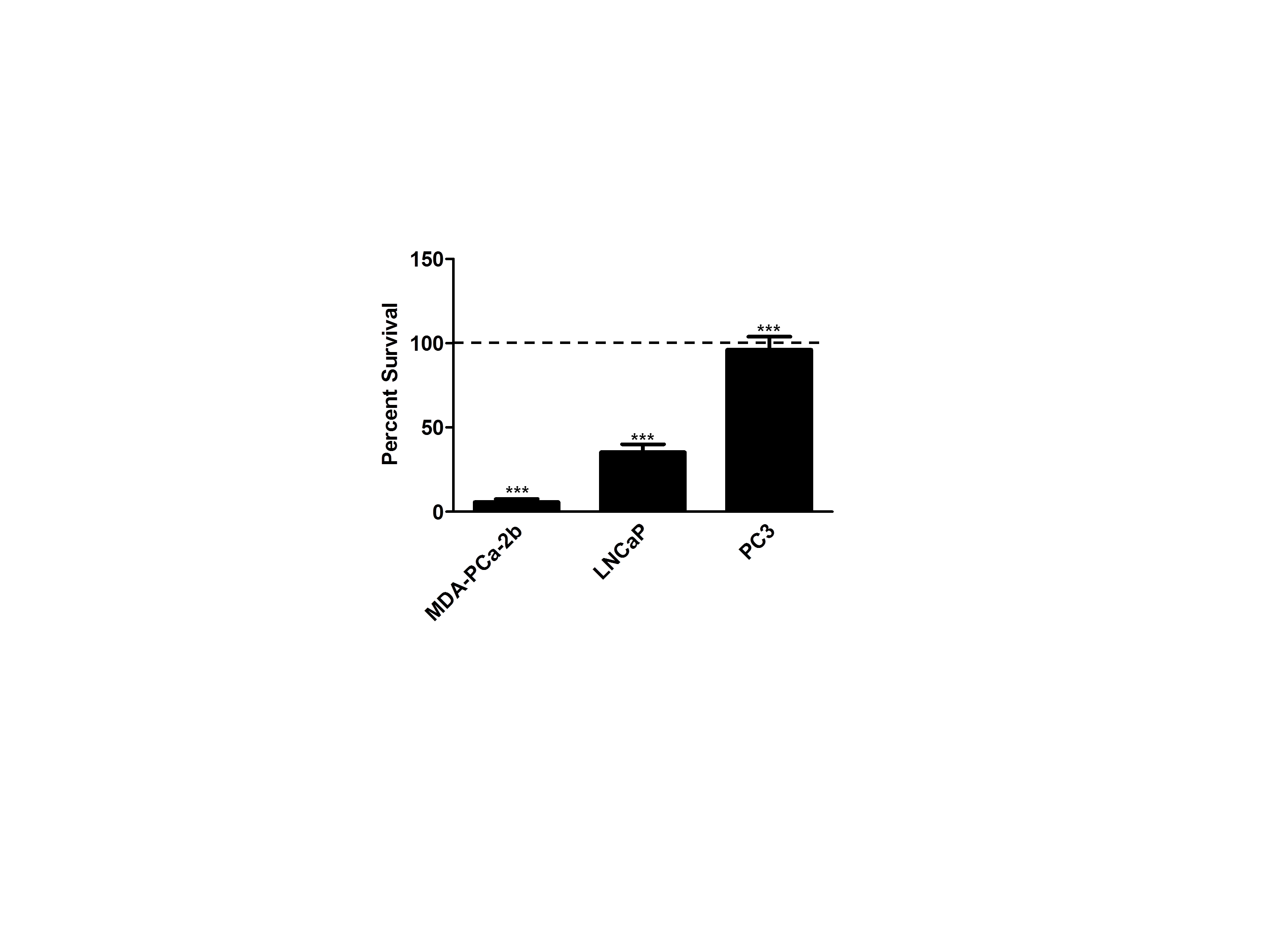

Supplement: Additional file 6: Figure S2 — Survival of PC3, LNCaP, and MDA-PCa-2b cells in androgen depleted media. Cell survival in androgen depleted conditions as compared to the normal growth media condition as measured with an MTT assay. One hundred percent is equal to the mean of that cell line in culture with its normal growth media. All pairs of groups were significant from each other at the 95% confidence interval (*** equals P-values < 0.001. [file 1471-2407-14-325-S6.tiff]

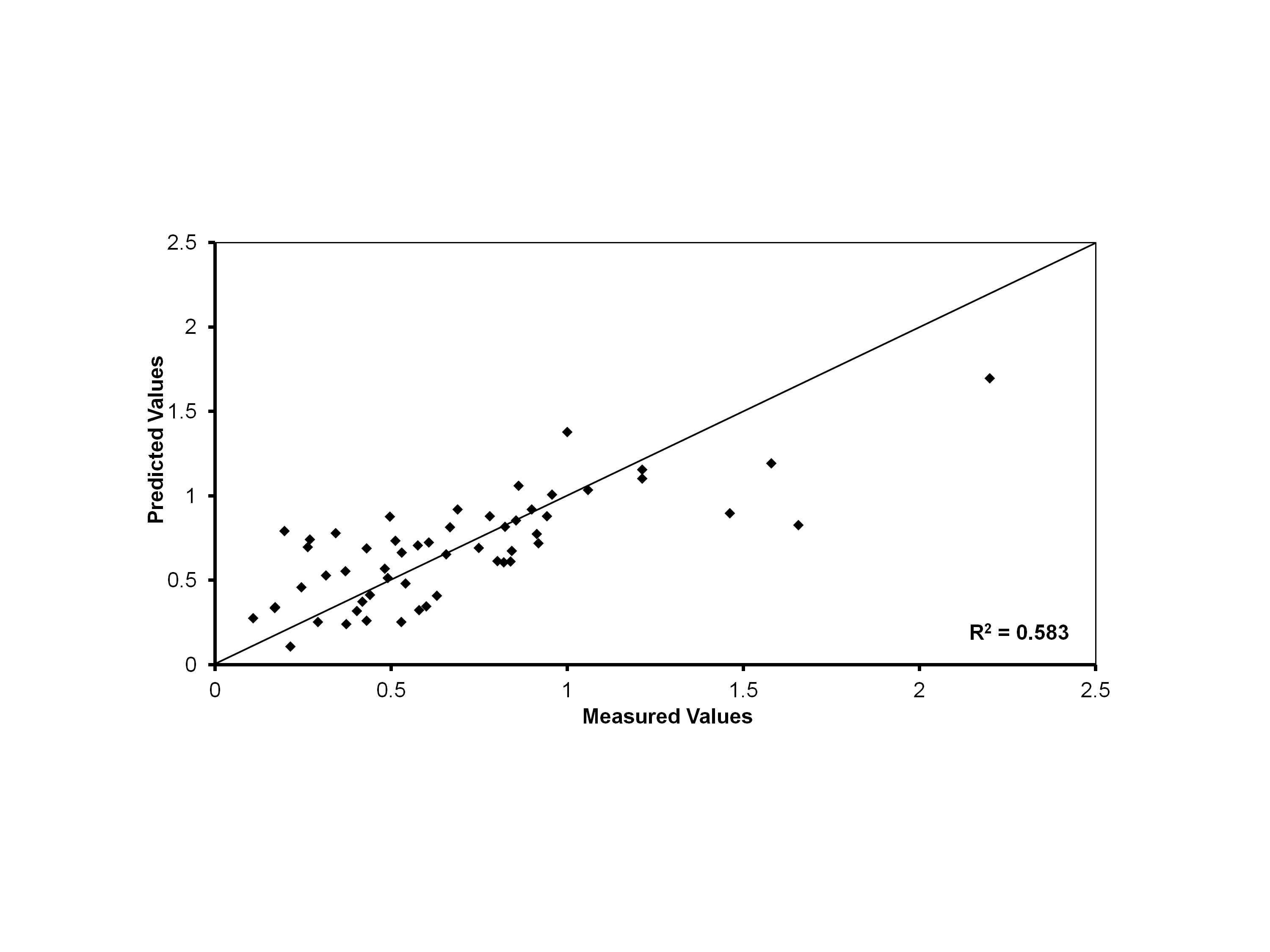

Supplement: Additional file 7: Figure S3 — The measured versus predicted survival of LNCaP cells. The predicted versus measured survival of LNCaP cells treated with targeted kinase inhibitors in combination with ligand treatments and docetaxel. A R2 value of 0.58 was calculated based on the partial least squares regression performed. [file 1471-2407-14-325-S7.tiff]

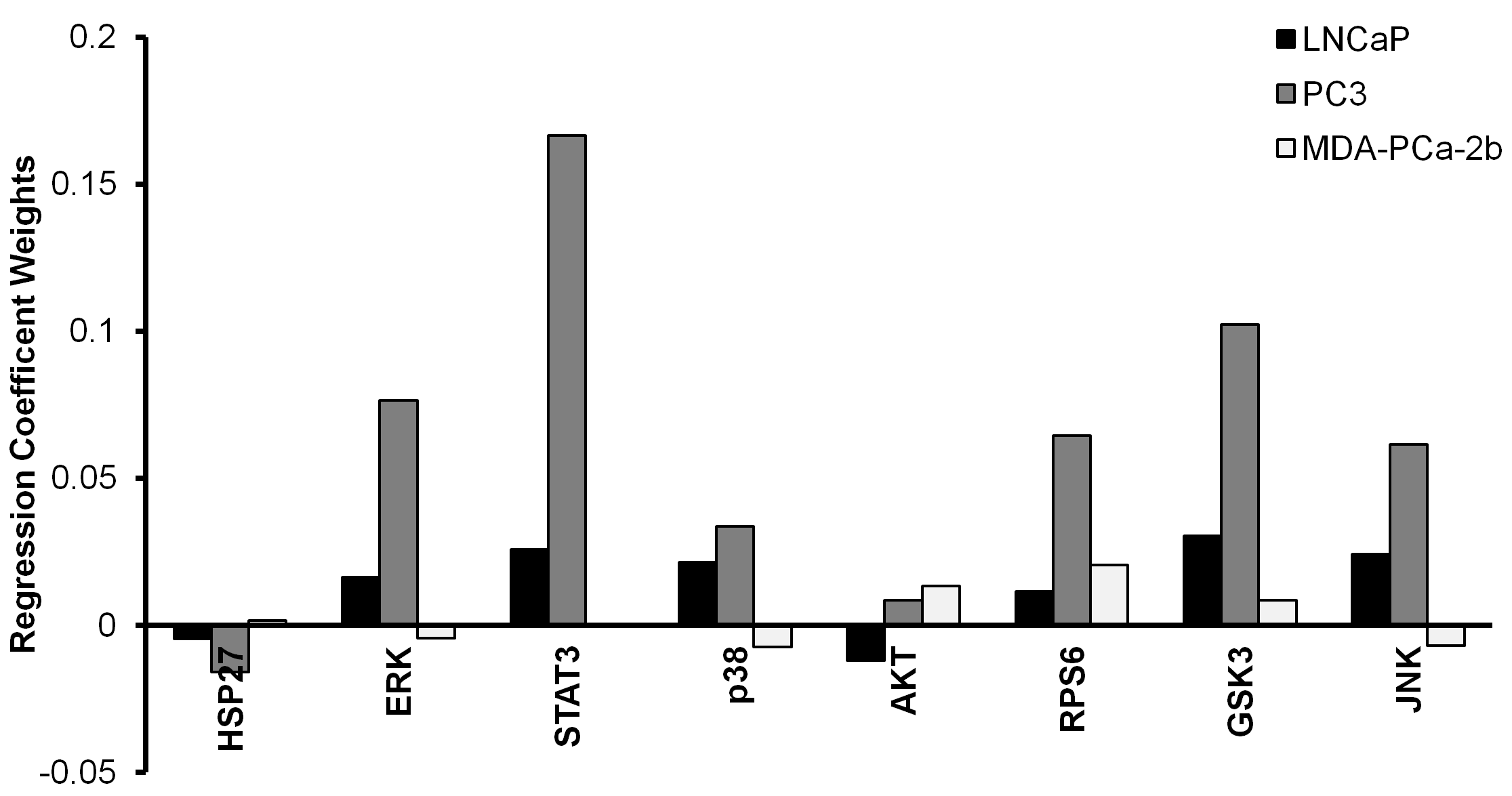

Supplement: Additional file 8: Figure S4 — The weights of the regression coefficients from PLSR for each cell line calculated individually. The regression coefficient’s weights for three different partial least squares regression models constructed on the data for the LNCaP, PC3, or MDA-PCa-2b cell lines when treated with EGF, IGF1, IL6, TNFα, DHT, and docetaxel. [file 1471-2407-14-325-S8.tiff]
